# Non-steroidal anti-inflammatory drugs among chronic kidney disease patients: an epidemiological study

**DOI:** 10.1186/s42506-018-0005-2

**Published:** 2019-01-30

**Authors:** Samar Abd ElHafeez, Reem Hegazy, Yasmine Naga, Iman Wahdan, Sunny Sallam

**Affiliations:** 1 Department of Epidemiology, High Institute of Public Health, Alexandria University, 165 ElHorreya Avenue, ElHadara, Alexandria, Egypt; 2 Ministry of Health, Alexandria, Egypt; 3 Department of Internal Medicine (Nephrology unit), Faculty of Medicine, Alexandria University, Alexandria, Egypt

**Keywords:** NSAIDs, CKD, Epidemiology

## Abstract

**Background:**

Non-steroidal anti-inflammatory drugs (NSAIDs) should be avoided among chronic kidney disease (CKD) patients. Till now, limited data are available on NSAID use in Egypt, and we aimed to study the prevalence and pattern of NSAID use among CKD patients.

**Methods:**

A cross-sectional study was done among 350 CKD adult patients presented to the Main Alexandria University Hospital. Those with end-stage renal disease and diagnosed with acute renal injury and pregnant women were excluded. Demographic and clinical data were collected by interviewing eligible patients. Data about the pattern, history of drug-drug interactions, and knowledge about the NSAID side effects were also gathered.

**Results:**

Of the enrolled patients, 57.1% were hypertensive, 46% were diabetics, 28% had osteoarthritis, and 18.3% had cardiovascular disease. CKD stages were 3.7%, 40.3%, and 56% in stages 2, 3, and 4, respectively. Almost two thirds (65.7%) were NSAID users. Among them, 82.6% were regular users. Headache was the most reported (68.7%) reason of use. The use of drugs which may have drug-drug interaction with the NSAIDs (as diuretics or renin-angiotensin-aldosterone system inhibitors) was reported in 36%. In multiple logistic regression, the odds of NSAID use decreased by 4% (odds ratio (OR) = 0.96, 95% confidence interval (CI) 0.93–0.99, *p* = 0.01) for every year increase in the patient’s age and decreased by 3% (OR = 0.97, 95% CI 0.95–0.99, *p* = 0.01) for every 1 ml/min/1.73 m^2^ increase in glomerular filtration rate.

**Conclusion:**

Despite the hazards of NSAID use on the kidney, still high proportion of CKD patients are using them for a long period and they are simultaneously using other drugs with possible drug-drug interactions. This study provided important information that would decrease the gap in knowledge about the use of NSAID in Egypt. It is recommended that NSAIDs should be used with caution among CKD patients and patients should be advised about its adverse health consequences.

## Introduction

Both over-the-counter and prescribed non-steroidal anti-inflammatory drugs (NSAIDs) are widely used all over the world. Although it is commonly used for the management of inflammation and pain, several guidelines including the Kidney Disease Initiative Global Outcome (KDIGO) guidelines recommended avoidance of NSAIDs (except aspirin and acetaminophen) for most patients with chronic kidney disease (CKD) [1].

The use of NSAIDs has been associated with renal function deterioration through variable mechanisms including alteration of the intraglomerular hemodynamic, nephrotic syndrome, glomerulonephritis, chronic interstitial nephritis, renal papillary necrosis, hyperkalemia, and podocyte injury [2–4]. This could lead to renal impairment and worsen the degree of renal dysfunction in CKD patients up to the development of end-stage renal disease (ESRD) [5]. Persons with CKD, however, are likely unaware of their disease and may also be unaware that NSAIDs should be avoided. Additionally, those with CKD are likely to be older and have multiple comorbid conditions or symptoms that lead to increased use of NSAIDs [6].

In addition, NSAIDs interact unfavorably with some commonly prescribed medications, including loop diuretics and renin-angiotensin-aldosterone system (RAAS) inhibitors. This is referred to as “triple whammy, leading to reduced effectiveness, along with increased risk of renal impairment. Although epidemiologic studies have linked NSAID use to progressive CKD, the risks of NSAIDs in patients with CKD, while supported by consensus and theoretical effect, remain less clearly established by evidence [7].

Despite the adverse effects of NSAIDs on renal functions, the available data on its pattern among CKD patients in Egypt is minimal. This study aimed to estimate the prevalence, to identify the pattern of NSAID use, and to assess the knowledge about their adverse effects in CKD patients. This will help in setting plans to reduce NSAID use in CKD patients, and spread awareness of their potential harms in this population.

## Materials and methods

### Study setting

The study was conducted in the outpatient clinics and the inpatient wards of the Internal Medicine Department, Alexandria Main University Hospital, which is the tertiary referral hospital for all patients from four governorates (Alexandria, El Beheira, Kafr-El Sheikh, and Marsa Matrouh).

### Study design

A cross-sectional study was carried out.

### Study population

Adult (18+ years) CKD patients diagnosed in pre-ESRD (i.e., before dialysis or transplantation) were included in the study. Patients < 18 years of age; those who were in stage 5 CKD (ESRD), on dialysis, with previous renal transplantation, and with acute renal injury; and pregnant women were excluded from the study.

### Sample size and sampling techniques

The sample size was determined using Epi-info software 7.2.2.6 (CDC, 2018) based on power 80%, with confidence level of 95% and prevalence of using NSAIDs among CKD patients of 65.8% [8]. The minimum required sample size was 340 patients. The sample was rounded to 350 patients. Patients were consecutively included on daily basis from the outpatient clinics and the inpatient wards until the required sample was reached.

### Data collection

A predesigned interview questionnaire was used to collect data from the patients about their personal characteristics (sociodemographic characteristics and smoking), history of comorbid diseases, history of selected drugs interacting with NSAIDs (For patients who were illiterate, the researchers had to show them the boxes of the drugs to know which one is taken.), and NSAID use including the type, purpose, pattern, and source of advice. In addition, knowledge about the adverse effects of NSAIDs was determined.

Concerning smoking, patients were classified into never smokers (those who have not smoked 100 cigarettes during their lifetime), current smokers (those who report smoking at least 100 cigarettes in their lifetime and who smoke cigarettes every day or some days), and former smokers (those who has smoked at least 100 cigarettes in their lifetime but does not smoke cigarettes) [9].

Blood pressure, weight, and height were measured, and standardized serum creatinine was collected from the patients’ records.

Regarding weight, patients were classified according to body mass index (BMI) [10] (weight in kilograms/height in meters^2^) into underweight (< 18.5 kg/m^2^), normal weight (18.5–24.9 kg/m^2^), overweight (25–29.9 kg/m^2^), and obese (≥ 30 kg/m^2^).

The estimated glomerular filtration rate (eGFR) was calculated by CKD-EPI equation [11]

$$ \mathrm{eGFR}=141\times \min {\left(\mathrm{Scr}/\kappa, 1\right)}^{\alpha}\times \max {\left(\mathrm{Scr}/\kappa, 1\right)}^{-1.209}\times {0.993}^{\mathrm{age}}\times 1.018\left[\mathrm{if}\ \mathrm{female}\right] $$

where Scr is serum creatinine (mg/dl), *ĸ* is 0.7 for females and 0.9 for males, *α* is − 0.329 for females and − 0.411 for males, min indicates the minimum of Scr/*ĸ* or 1, and max indicates the maximum of Scr/*ĸ*.

CKD was defined according to the 2012 KDIGO guidelines [1] according to the presence of the following criteria for more than 3 months:

1. Markers of kidney damage (one or more)
  Albuminuria (AER ≥ 30 mg/24 h; ACR ≥ 30 mg/g [≥ 3 mg/mmol])
  Urine sediment abnormalities
  Electrolyte and other abnormalities due to tubular disorders
  Abnormalities detected by histology
  Structural abnormalities detected by imaging
  History of kidney transplantation
2. Decreased GFR (GFR < 60 ml/min/1.73 m^2^ (GFR categories G3a–G5))

CKD is classified into the following stages:

- G1: normal or high GFR (> 90 ml/min/1.73 m^2^)
- G2: mild decrease in GFR (60–89 ml/min/1.73 m^2^)
- G3a: mild to moderate decrease in GFR (45–59 ml/min/1.73 m^2^)
- G3b: moderate to severe decrease in GFR (30–44 ml/min/1.73 m^2^)
- G4: severe decrease in GFR (15–29 ml/min/1.73 m^2^)
- G5: kidney failure (GFR < 15)

We excluded patients in CKD stage 5 as those patients already reached ESRD and so prevention of NSAID use will not add any benefits to the disease progression.

### Statistical analysis

Data was summarized using mean ± SD, medians and inter-quartile ranges or frequencies and percentage, as appropriate. Comparison between the variables was done using *t* test, Mann-Whitney or chi-square according to the data type. Correlation between the renal function and duration of NSAID use was done by Pearson’s correlation. The factors associated with the use of NSAIDs were identified using multiple logistic regression analysis. The model included all variables which were significantly related to NSAID use in bivariate analysis. Results are considered significant when *p* < 0.05.

## Results

Table 1 summarizes the baseline characteristics of the study patients. The mean ± SD age of the study participants (*N* = 350) was 55 ± 13 years, 51% were males, 74.5% were married, 40.8% were smokers, 42% were obese, 57.1% were hypertensive, 46% were diabetics, 18.3% had cardiovascular disease, and 4.9% had autoimmune disease. More than half (56%) of the patients were in stage 4 CKD, and 40.3% were in stage 3 CKD. One fifth (20%) of the patients gave history of RAAS inhibitor use, 9.7% were using diuretics, and 6.3% reported the use of both.

**Table 1:** Characteristics of the study CKD patients, Alexandria Main University Hospital, Egypt, 2016

Characteristics	Total (*n* = 350) No. (%)	NSAID users (*n* = 230) No. (%)	NSAID non-users (*n* = 120) No. (%)	*p* value
I. Personal characteristics				
Age (years) Mean ± SD	55 ± 13	54 ± 13	57 ± 13	0.06
Gender				
Male	180 (51.4)	105 (45.7)	75 (62.5)	0.003*
Female	170 (48.6)	125 (54.3)	45 (37.5)	
Marital status				
Unmarried	17 (4.9)	12 (5.2)	5(4.2)	
Married	261 (74.5)	169 (73.5)	92(76.7)	0.64
Else	72(20.6)	49(21.3)	23(19.1)	
Education				
Illiterate	223(63.7)	136 (59.2)	87 (72.5)	
School education	116 (33.1)	86 (37.3)	30 (25.0)	0.13
University	11 (3.2)	8 (3.5)	3 (2.5)	
Occupation				
Not working	206 (58.8)	145 (63)	61 (50.8)	
Clerk	29 (8.3)	23 (10)	6 (5)	0.003*
Worker	42 (12)	27 (11.7)	15 (12.5)	
Retired	73 (20.9)	35 (15.3)	38 (31.7)	
Smoking status				
Never smoker	207 (59.2)	146 (63.5)	61 (50.8)	0.02*
Current smoker	67 (19.1)	44 (19.1)	23(19.2)	
Former smoker	76 (21.7)	40 (17.4)	36 (30)	
Weight categories				
Normal	54 (15.4)	34 (14.8)	20 (16.7)	0.85
Overweight	149 (42.6)	100 (43.5)	49 (40.8)	
Obese	147 (42)	96 (41.7)	51 (42.5)	
II. Comorbid diseases				
Hypertension				
Yes	200 (57.1)	146 (63.5)	54 (45)	0.001*
No	150 (42.9)	84 (36.5)	66 (55)	
Diabetes				
Yes	161 (46)	105 (45.7)	56 (46.7)	0.86
No	189 (54)	125 (54.3)	64 (53.3)	
Osteoarthritis				
Yes	98 (28)	82 (35.7)	16 (13.3)	< 0.001*
No	252 (72)	148 (64.3)	104 (86.7)	
Autoimmune disease (RA and SLE)				
Yes	17 (4.9)	15 (6.5)	2 (1.7)	0.05
No	333 (95.1)	219 (93.5)	118 (98.3)	
CVDs				
Yes	64 (18.3)	42 (18.3)	22 (18.3)	0.99
No	286 (81.7)	188 (81.7)	98 (81.7)	
Hepatitis C virus				
Yes	58 (16.6)	23 (10)	35 (29.2)	< 0.001*
No	292 (83.4)	207 (90)	85 (70.8)	
Cancer				
Yes	13 (3.7)	11 (4.8)	2 (1.7)	0.23
No	337 (96.3)	219 (95.2)	118 (98.3)	
Kidney stone				
Yes	9 (2.6)	7 (3)	2 (1.7)	0.72
No	341 (97.4)	223 (97)	118 (98.3)	
III. Chronic kidney disease				
eGFR (ml/min/1.73 m^2^)				
Median (inter-quartile range)	27 (18–37)	26 (18–34)	31 (19–44.75)	0.003*
CKD stage				
Stage 2	13 (3.7)	6 (2.6)	7 (5.8)	
Stage 3a	44 (12.6)	21 (9.1)	23(19.2)	0.007*
Stage 3b	97 (27.7)	62 (27)	35 (29.2)	
Stage 4	196 (56.0)	141 (61.3)	55 (45.8)	
IV. History of intake of selected interacting drugs with NSAIDs				
Diuretics	34 (9.7)	19 (8.3)	8 (6.7)	0.20
RAAS inhibitors	70 (20)	53 (23)	17 (14.2)	0.04*
Both	22 (6.3)	14 (6.1)	8 (6.7)	0.83

*SD* standard deviation, *RA* rheumatoid arthritis, *SLE* systemic lupus erythmatosus, *CVDs* cardiovascular diseases, *eGFR* estimated glomerular filtration rate, *CKD* chronic kidney disease, *RAAS* renin-angiotensin-aldosterone inhibitors, *NSAIDs* non-steroidal anti-inflammatory drugs

*Significant at *p* < 0.05

### Prevalence and pattern of NSAID use

**Fig. 1:**
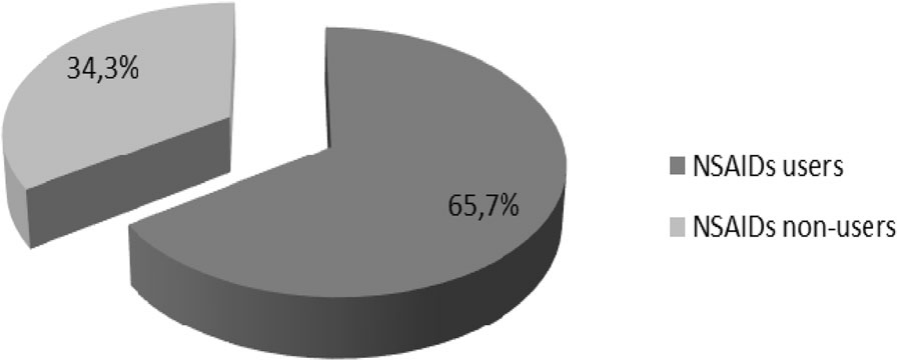
Prevalence of NSAID use among the study CKD patients

About two thirds (65.7%) of the study patients reported NSAID use (Fig. 1). Patients who use NSAIDs were younger, females, non-smokers, and hypertensive; had osteoarthritis; and were more users of RAAS inhibitors (Table 1).

**Fig. 2:**
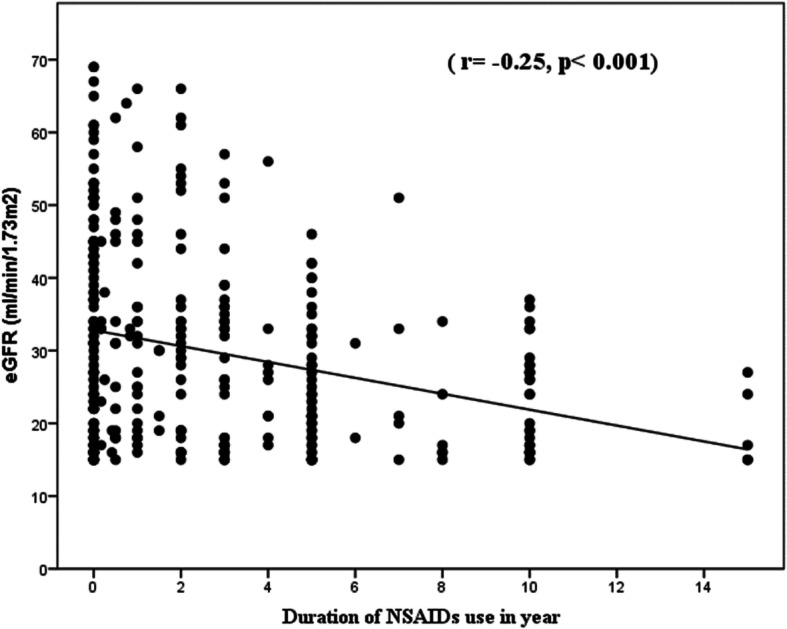
Correlation between the duration of NSAID use and eGFR

Among NSAID users, ketoprofen was the most commonly used (50.3%), followed by diclofenac (33.7%), ibuprofen (23.1%), ketorolac (2.6%), and meloxicam (2.3%) (Table 2). More than two thirds (68.7%) of the patients reported headache as the main reason of NSAID use, followed by generalized pain (49.6%) and joint pain (43.9%). Forty percent of NSAID users used NSAIDs twice a week, 20.4% three times a week, and 22% every day within the last month. More than 80% (82.6%) of NSAID users gave history of regular use (at least twice a week for more than 2 months). More than 40% of the NSAID users used NSAIDs for a period exceeding 3 years or from 1 to 3 years (46.1% and 41.7%, respectively) (Table 2). There was an inverse correlation between the eGFR and the duration of NSAID use (*r* = − 0.25, *p* < 0.001) (Fig. 2).

**Table 2:** NSAID use by the study CKD patients (Alexandria Main University Hospital, Egypt, 2016)

NSAID use	Frequency (*n* = 230)	Percent
Types of NSAIDs^a^		
Ketoprofen	176	50.3
Diclofenac	118	33.7
Ibuprofen	81	23.1
Ketorolac	9	2.6
Meloxicam	8	2.3
Purpose of use^a^		
Headache	158	68.7
Generalized pain	114	49.6
Joint pain	101	43.9
Renal colic	26	11.3
Dental pain	23	10.0
Menstrual pain	3	1.3
Pattern of use		
Frequency of NSAID use within last month		
Once a week	40	17.4
Twice a week	92	40.0
Three times a week	47	20.4
Every day	51	22.2
Duration of NSAID use		
< 1 year	28	12.2
1–3 years	96	41.7
> 3 years	106	46.1
Regular use		
Yes	190	82.6
No	40	17.4
Number of NSAIDs used within last month		
One type	103	44.8
Two types	92	40.0
Three types	31	13.5
More than three	4	1.7
Route of administration within last month		
Oral	171	74.3
Injection	2	0.9
Both	57	24.8

*NSAIDs* non-steroidal anti-inflammatory drugs

^a^Non-mutually exclusive

The majority of NSAID users (76.5%) used NSAIDs by self-decision, while 25.2% used them after the advice of physicians. Those who mentioned relatives and friends constituted 13.5%. Pharmacists and previous prescription constituted 10.4% and 9.1%, respectively.

### Knowledge about adverse effects of NSAIDs

More than half (53.2%) of CKD patients did not know whether NSAIDs have adverse effects or not. Those who mentioned that NSAIDs have adverse effects constituted 37.1%, and only 9.7% said that NSAIDs have no adverse effects. Among CKD patients who mentioned that NSAIDs have adverse effects, 55.4% thought that NSAIDs cause kidney problems. The patients who mentioned that NSAIDs lead to gastrointestinal tract, liver problems, and heart problems constituted 45.4%, 19.2%, and 2.3%, respectively (Table 3).

**Table 3:** Knowledge of CKD patients about adverse effects of NSAIDs (Alexandria Main University Hospital, Egypt, 2016)

Knowledge about adverse effects	Frequency (*n* = 350)	Percent
NSAIDs have adverse effects	34	9.7
No	130	37.1
Yes	186	53.2
Do not know		
Type of adverse effects (*n* = 130)^a^		
Kidney problems		
GIT problems	72	55.4
Liver problems	59	45.4
Do not know	25	19.2
Heart problems	13	10.0
	3	2.3

*GIT* gastrointestinal tract

^a^Non-mutually exclusive

### Factors affecting NSAID use among CKD patients

Multiple logistic regression analysis of the factors affecting NSAID use among CKD patients revealed that NSAID use significantly decreased with increase in the age of the patients and increase in eGFR and those among hepatitis C patients. On the contrary, NSAID use was significantly higher among working, hypertensive, and osteoarthritis patients (Table 4).

**Table 4:** Multiple logistic regression analysis for the factors affecting NSAID use among CKD patients, Alexandria Main University Hospital, 2016

Independent variable	Unit of the variable	OR	95% CI		*p* value
			Lower limit	Upper limit	
Age	1 year	0.96	0.93	0.99	0.01*
Gender	0 = female, 1 = male	1.07	0.26	4.43	0.93
Working status	0 = not working, 1 = working	2.66	1.19	5.95	0.02*
Smoking	0 = non-smoker, 1 = smoker	1.03	0.43	2.51	0.94
eGFR	1 ml/min/1.73 m^2^	0.97	0.95	0.99	0.01*
Hypertension	0 = no, 1 = yes	2.60	1.27	5.35	0.008*
Osteoarthritis	0 = no, 1 = yes	6.34	2.03	19.78	< 0.001*
Hepatitis C	0 = no, 1 = yes	0.15	0.06	0.40	< 0.001*

*Significant at *p* < 0.05

## Discussion

In this cross-sectional study of 350 pre-ESRD patients recruited from the Alexandria Main University hospital, the prevalence of NSAID usage was 65.7%. There was a gradient increase in NSAID use through CKD stages with a prevalence of 2.6% in stage 2, 9.1% in stage 3a, 27% in stage 3b, and 61.3% in stage 4. Ketoprofen was the most commonly used drug. In addition, prolonged use of NSAIDs was related to reduction of eGFR.

The use of NSAIDs has been reported to range between 8.9 and 69.2% [8–13]. Variations in the NSAID use in many studies could be explained by differences in the regulations on NSAID purchase and its availability in different countries, with the absence of restricted laws on the consumption of drugs, which encourages patients to self-treat their symptoms and signs, especially pain [14, 15]. Moreover, the silent nature of CKD and unawareness of the NSIAD complications predispose to the late diagnosis, with the inappropriate use of drugs [16, 17]. In addition, the high use of NSAIDs may indicate that clinicians tend to disregard evaluating the renal functions of patients when prescribing NSAIDs especially in high-risk group patients or they may want to achieve a better quality of life in some comorbid conditions warranting the use of NSAIDs despite the inherent risk.

Consistent with reports from other studies [9, 18, 19], headache was the most reported reason of NSAID use, followed by generalized pain and joint pain. Renal patients can experience pain as a result of several conditions and pathophysiological mechanisms. Osteodystrophy, osteoarthritis, calciphylaxis, and peripheral neuropathy may develop as consequence of the progressive course of CKD. Pain is also related to comorbidities such as peripheral vascular disease, cardiovascular disease, diabetic neuropathy, osteoporosis/osteopenia, and inflammatory/immunological diseases [20–22]. Their need for analgesia presents a serious challenge to clinicians and requires special considerations to achieve pain relief without causing toxicity [21].

Although it has been known that NSAIDs inhibit the therapeutic effect of RAAS inhibitors and diuretics (triple whammy), it was noted from the current study that many CKD patients who used NSAIDs were also receiving RAAS inhibitors (23%), diuretics (8.3%), or both (6.1%). A similar problem was reported by Plantinga et al. in the USA [23]. They found that among CKD patients who had prescriptions for NSAIDs, 16% had RAAS inhibitor prescriptions and 20% had diuretic prescriptions.

The pattern of NSAIDs in the current study showed that 40% of CKD patients used NSAIDs twice a week, 20.4% three times a week, and 22% every day. A considerable percentage used NSAIDs for a period exceeding 3 years or from 1 to 3 years (46.1% and 41.7% respectively). Only 12.2% of CKD patients used NSAIDs for less than 1 year. In other studies, the pattern of NSAID use was different. In Poland (2016) [18], 12.2% of CKD (stages 1–4) used NSAIDs few times a week, 14.8% few times a month, and 7.7% every day. In Southern Italy (2014) [13], more than one third (35.6%) of CKD patients were treated with NSAIDs for a period exceeding 90 days and almost 16.5% for more than 6 months. In USA (2011), Plantinga et al. [23] showed that 65% of CKD (stages 1 or 2) and 64.4% of CKD (stages 3 or 4) were long-term users of NSAIDs (1 year or longer).

A significant association between eGFR and NSAID use was found in the present study as the odds of NSAID use decreased by 3% for every 1 ml/min/1.73 m^2^ increase in eGFR. This association is consistent with what was reported in several studies. Senevirathna et al. [24] conducted a cohort study among 143 CKD patients of uncertain etiology to determine the possible factors associated with the progression and mortality of CKD. They reported that NSAIDs were a major individual factor for disease progression. Kuo and his colleagues [25] carried out a cohort study using a nationally representative sample of 19,163 newly diagnosed CKD patients to study the risk of various analgesics use on the progression of CKD. CKD patients using non-selective NSAIDs had an increased risk of CKD stage 5. The trends toward higher risk with increasing exposure dose were significant for all classes of analgesics.

### Limitations of the study

Our study has limitations. First, it is a cross-sectional study, so we were not able to study the causal association between NSAID use and CKD progression. Second, our main definition did not include the most commonly used over-the-counter analgesics—acetaminophen and aspirin—because of their relatively low nephrotoxicity and indications for pain and cardiovascular prevention, respectively. Third, participants with chronic pain may over- or underestimate the duration and frequency of NSAID use. This could be due to high illiteracy rate. In addition, we did not have information on clinician recommendation, or detailed dosage and frequency. Fourth, there is the possibility of CKD misclassification, particularly for earlier stages. Finally, information on comorbid conditions was taken by clinical history.

## Conclusion

As there are no available previous studies on the use of NSAID in kidney disease patients in Egypt, this study provided important information that would decrease the gap in knowledge about the use of NSAID in Egypt. It also has important implications. It reflects the importance of communicating the effects of NSAID use especially their nephrotoxicity and potential interactions with RAAS inhibitors and diuretics to physicians, other prescribers of medication, and the community through training courses and workshops.
